# Volume changes in the contralateral submandibular gland following unilateral gland excision in oral cancer patients

**DOI:** 10.1186/s40902-024-00446-5

**Published:** 2024-10-11

**Authors:** Yei-Jin Kang, Young-Wook Park, Hang-Moon Choi, Seong-Gon Kim

**Affiliations:** 1 Department of Oral and Maxillofacial Surgery, College of Dentistry, Gangneung-Wonju National University, Gangneung, 28644 Republic of Korea; 2 Department of Oral and Maxillofacial Radiology, College of Dentistry, Gangneung-Wonju National University, Gangneung, 28644 Republic of Korea

**Keywords:** Submandibular gland excision, Compensatory hypertrophy, Oral cancer, Salivary gland volume

## Abstract

**Background:**

The effects of unilateral submandibular gland excision on the size of the contralateral gland are not well understood, with no human studies reported to date. This study aims to investigate the impact of unilateral submandibular gland excision on the contralateral gland’s size, providing insights into compensatory mechanisms and their clinical implications.

**Method:**

This retrospective study involved patients with oral cancer who underwent unilateral submandibular gland excision and ipsilateral neck dissection at Gangneung-Wonju National University Dental Hospital between 2008 and 2023. Patients were included if they had preoperative and follow-up 3D radiological images. The contralateral submandibular gland volume was measured using 3D Slicer software on preoperative, post-operative, and follow-up radiographic data.

**Results:**

The mean volume change of the contralateral submandibular gland was 1.35 ± 2.06 cm3, with a mean change ratio of 1.18 ± 0.24. These changes were statistically significant (*p* = 0.006). Other factors such as age, gender, and radiotherapy did not significantly affect the volume change ratio (*p* > 0.05).

**Conclusion:**

The contralateral submandibular gland exhibits a statistically significant increase in volume following unilateral gland excision, indicating compensatory hypertrophy. This morphological adaptation should be considered in post-operative care and surgical planning for oral cancer patients to optimize outcomes.

## Background

The submandibular gland is one of the major salivary glands and is frequently removed during surgery due to conditions such as inflammation, benign tumors, or cancer [1]. Many patients undergoing submandibular gland removal are elderly and may have other medical conditions, although this procedure is also performed in younger individuals depending on the underlying condition. The removal of a major salivary gland can significantly impact the oral environment and quality of life [2].

Xerostomia, characterized by a reduced or absent flow of saliva, poses challenges for affected individuals, impacting their oral health, nutrition, and overall quality of life [3]. Xerostomia can arise not only as a side effect of radiotherapy (RT) but also because of surgical removal of a salivary gland. The submandibular gland, responsible for approximately 70% of unstimulated saliva, plays a crucial role in contributing to about 95% of total daily saliva production [4]. As such, its removal can lead to significant functional impairment, particularly in the absence of compensatory secretion from the remaining salivary glands.

While some studies have explored the volumetric changes in salivary glands following the removal of the contralateral gland [5], research on this topic remains limited. For instance, one study in the rats reported compensatory hypertrophy in the contralateral salivary gland after resection of the opposite gland, noting a 10% increase in cell size and a 17% increase in nucleus size [6]. In a study on mice, the area of the acini in the remaining submandibular gland significantly increased 21 days after unilateral salivary gland resection. This suggests that acinar cells began proliferating as early as 7 days after the resection, leading to an increased acinar area and submandibular gland weight by day 21 [7]. In a study on rats, cellular changes in the hyperplastic submandibular gland suggest the death of newly generated acinar cells and the expansion of the gland’s progenitor cell compartment, indicated by the elongation of the intercalated ducts [8]. In human studies, salivary flow decreased after treatment but recovered after more than 3 years. However, it was not clarified whether the salivary gland was removed or not, and the effect of adjuvant therapy may have been significant. Additionally, no volumetric analysis of the glands was performed [9].

Despite these observations, the effects of unilateral submandibular gland excision on the size of the contralateral gland remain poorly understood. To our best knowledge, no human studies have been reported on volumetric analysis of the salivary glands. Therefore, the aim of this study is to investigate the impact of unilateral submandibular gland excision on the contralateral gland’s size. By doing so, this study seeks to provide valuable insights into the compensatory mechanisms involved and their potential clinical implications.

## Methods

### Study design

This retrospective study was approved by the Institutional Review Board of Gangneung-Wonju National University Dental Hospital (GWNUDH-IRB2024-A004). The study included patients diagnosed with oral cancer who underwent unilateral submandibular gland excision and ipsilateral neck dissection at Gangneung-Wonju National University Dental Hospital between 2008 and 2023. The study population was identified through a review of hospital records, and all included patients had preoperative and follow-up 3D radiological imaging available for analysis.

### Data collection

**Fig. 1:**
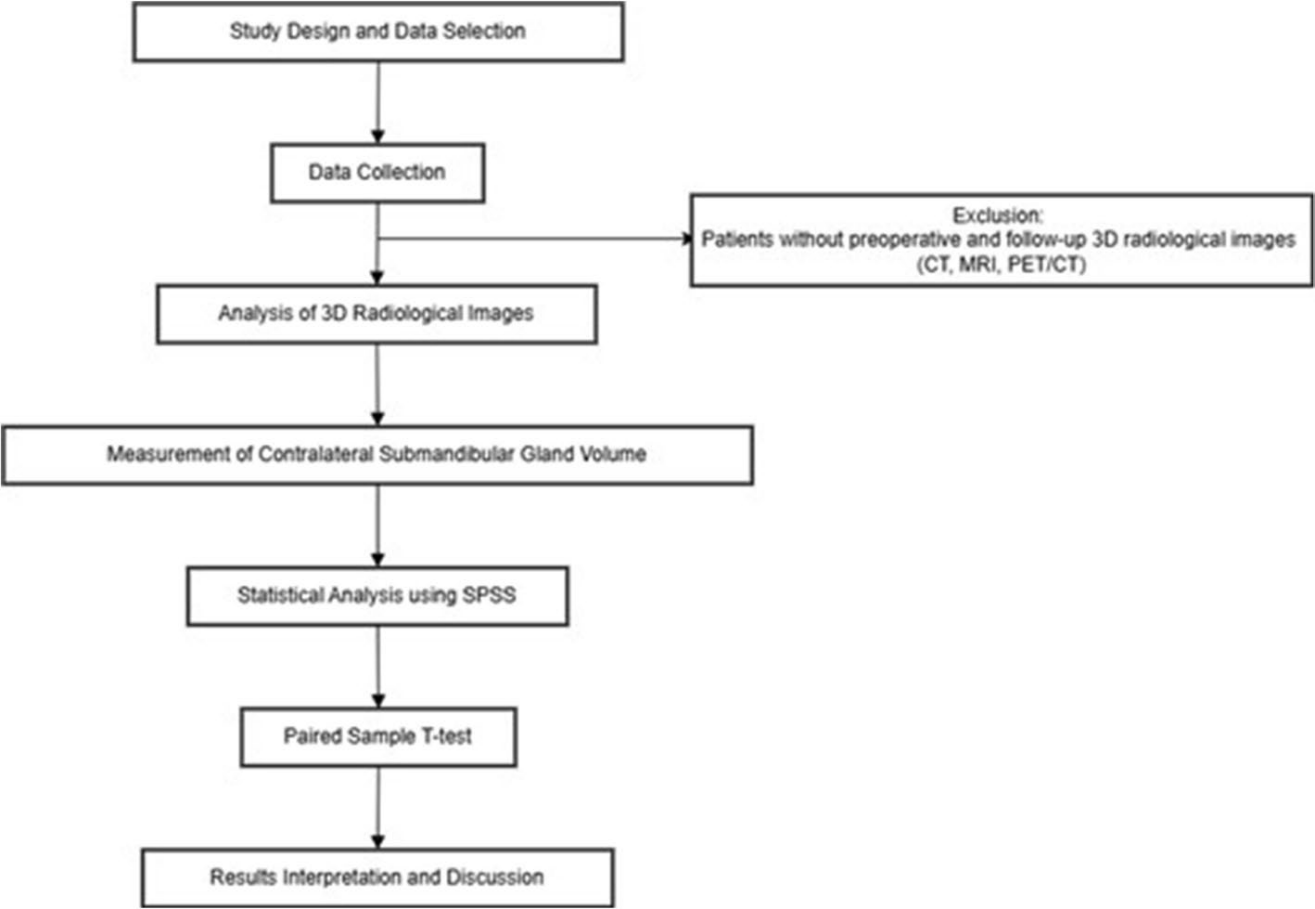
Flow diagram of the study

**Fig. 2:**
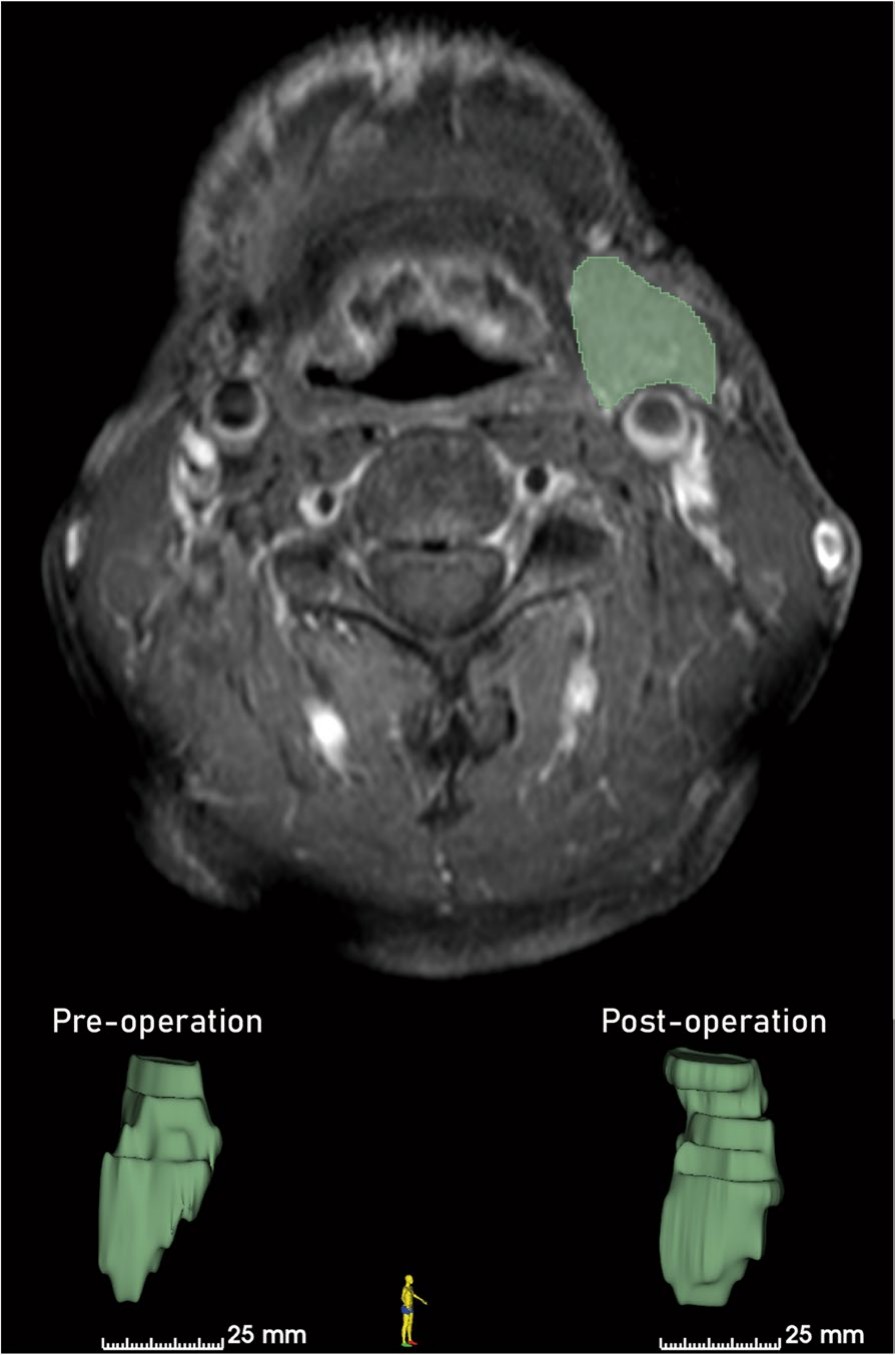
Volumetric analysis of the contralateral submandibular gland using 3D Slicer software (version 5.6.2). The volume measurements were performed on preoperative, immediate post-operative, and follow-up 3D radiographic data. The segmentation of the submandibular gland was manually delineated slice by slice (upper image), and the software calculated the total gland volume based on these segmentations (lower left and right images)

A comprehensive review of the patients’ surgical reports, pathological findings, and radiological images was conducted (Fig. 1). Inclusion criteria required patients to have complete preoperative and follow-up 3D imaging data (CT, MRI, or PET/CT) that allowed for accurate volumetric analysis (Fig. 2). Patients were excluded if they lacked either preoperative or follow-up 3D images or if the imaging data was of insufficient quality for volumetric analysis.

### Analysis of 3D radiological images

The contralateral submandibular gland volume was measured using 3D Slicer software (version 5.6.2), a free, open-source platform for medical image analysis. The volume measurements were performed on preoperative, immediate post-operative, and follow-up 3D radiographic data. The submandibular gland is in the posterior part of the submandibular triangle, which is bordered by the body of the mandible superiorly, the anterior belly of the digastric muscle medially, and the posterior belly of the muscle inferiorly and laterally. The gland is enclosed by a capsule that is part of the superficial layer of the deep cervical fascia. Like the parotid gland, the submandibular gland is divided into superficial and deep lobes, which are separated by the mylohyoid muscle. The larger superficial lobe lies beneath the deep cervical fascia [10]. The landmark to discern the submandibular gland was identified on axial images by its anatomical position and its distinctive U-shape as it slightly overlaps the mylohyoid muscle. The segmentation of the submandibular gland was manually delineated slice by slice, and the software calculated the total gland volume based on these segmentations. To ensure accuracy, measurements were performed by two independent radiologists, and the mean volume was used in the final analysis.

### Statistical analysis using SPSS

Statistical analyses were conducted using SPSS software (version 28.0.0.0, SPSS Inc., Chicago, IL, USA). The normality of the data was assessed using the Kolmogorov–Smirnov test. To evaluate the change in contralateral submandibular gland volume between preoperative and follow-up imaging, a paired-sample *t*-test was performed. The significance level was set at *p* < 0.05. Additionally, subgroup analyses were conducted to assess the impact of variables such as age, gender, and adjuvant radiotherapy on gland volume changes.

## Results

As a result of a retrospective study, 22 patients (16 men and 6 women) with oral cancer, who underwent excision and ipsilateral neck dissection in Gangneung-Wonju National University Dental Hospital were included (Tables 1 and 2). Of the 22 patients, 7 patients underwent adjuvant radiotherapy.

**Table 1:** Descriptive statistics

Variables		Value
Gender	Male	16
	Female	6
Age		67.05 ± 9.11 years
Operation side	Left	12
	Right	10
Post-operative radiotherapy	No	15
	Yes	7
Pathology	Squamous cell carcinoma	18
	Verrucous carcinoma	1
	Basal cell carcinoma	1
	Adenoid cystic carcinoma	1
	Mucoepidermoid carcinoma	1

**Table 2:** Pathologic stage and neck dissection level

Case	Stage	Neck dissection level
1	pT2N0M0, Stage II	Functional ND Lv.Ib, V
2	pT2N0M0, Stage II	SOHND Lv.I, II, III
3	pT2N0M0, Stage II	SOHND Lv. I
4	pT2N0M0, Stage II	SOHND Lv.I, II, III
5	pT2N0M0, Stage II	SOHND Lv. I
6	pT2N0M0, Stage II	SOHND Lv.I, II, III
7	pT2N2bM0, Stage IVA	SOHND Lv.I, II, III
8	pT2N0M0, Stage II	SOHND Lv.I, II
9	(recurrent)pT2N0M0, Stage II	SOHND Lv.I, II
10	pT4aN0M0, Stage IVA	SOHND Lv. I, II
11	pT2N2bM0, Stage IVA	SOHND Lv. I, II
12	pT2N0M0, Stage II	SOHND Lv. I, II, III
13	pT2N0M0, Stage II	SOHND Lv. I
14	pT2N2bM0, Stage IVA	SOHND Lv.I, II, III, IV
15	pT2N0M0, Stage II	SOHND Lv. I, II, III
16	pT4aN0M0, Stage IVA	SOHND Lv. I, II
17	pT1N0M0, Stage I	SOHND Lv. I
18	pT4aN0M0, Stage IVA	SOHND Lv. I
19	pT4aN2bM0, Stage IVA	SOHND Lv. I, II, IV
20	pT2N1M0, Stage III	SOHND Lv. I, IIa
21	pT4aN0M0, Stage IVA	SOHND Lv. I, II, III
22	pT4aN0M0, Stage IVA	SOHND Lv. I, II

*Lv* level, *ND* neck dissection, *SOHND* supra-omohyoid neck dissection

**Fig. 3:**
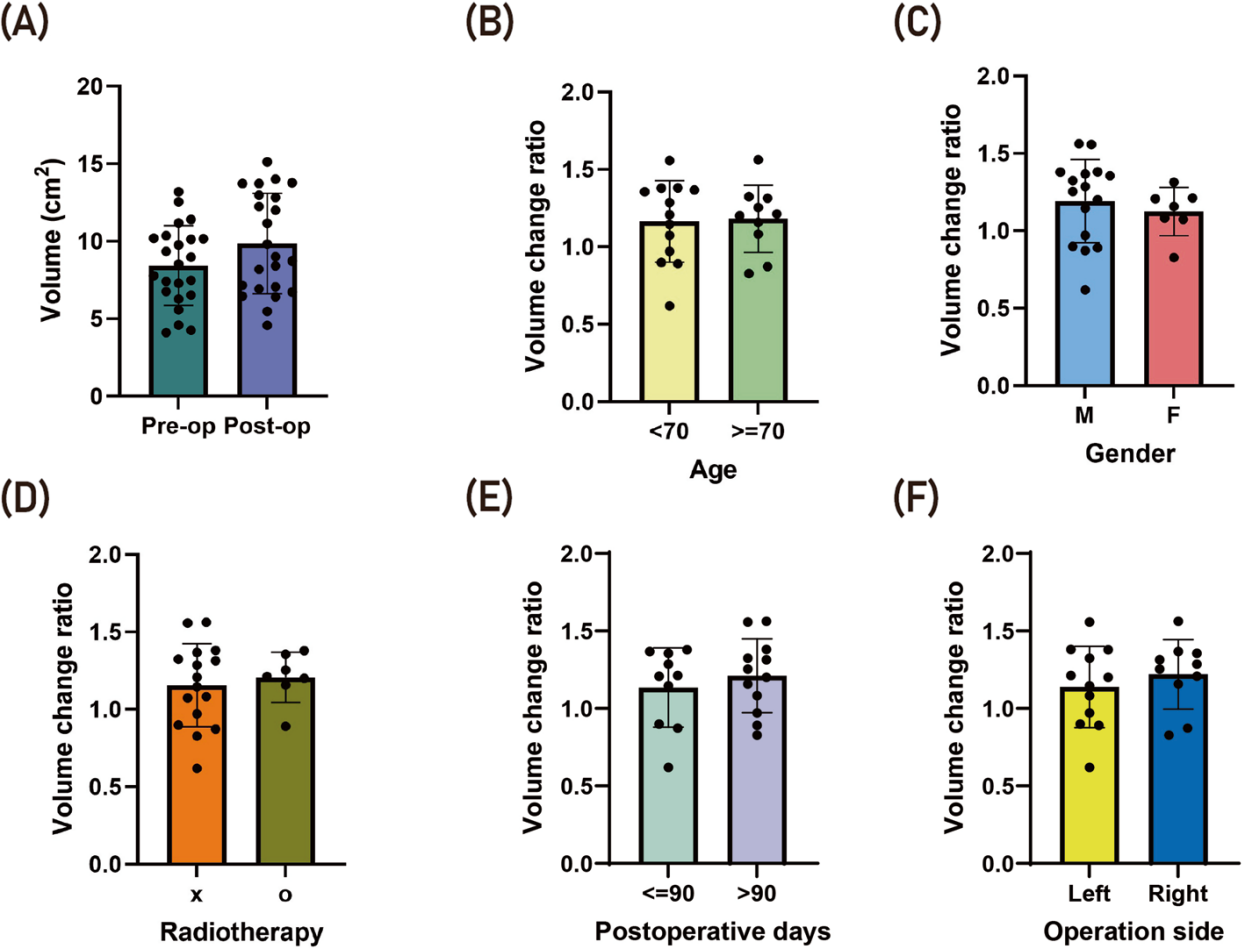
**A** The size of the contralateral submandibular gland was significantly increased post-operatively after the ipsilateral submandibular gland extirpation. **B** There was no significant difference in post-operative volume change between the groups aged over 70 and those under 70. Younger patients may have a greater capacity for glandular compensation due to more robust cellular regeneration and overall physiological resilience. **C** The volume change ratio in males was larger than that in females; however, the difference between the groups was not statistically significant. **D** There was no significant difference in post-operative salivary gland volume change by the presence of post-operative radiation therapy in this study

The contralateral submandibular gland volume changes between preoperative and post-operative were represented in Fig. 3. The average post-operative days when the post-operative image was taken was 152.5 days (minimum 30 days, maximum 840 days). The mean volume change ratio was compared by dividing the post-operative observation point in under 3 months and over 3 months. Mean and standard deviation were calculated. The mean volume of the contralateral submandibular gland was 8.74 ± 2.46 cm^3^ and 10.09 ± 3.11 cm^3^ in pre- and post-operatively respectively (Fig. 3A). When compared both groups, the difference was statistically significant (*p* = 0.006). The mean volume increase after operation was 1.35 ± 2.06 cm^3^. The mean volume change ratio was 1.17 ± 0.24.

The mean volume change ratio in the older group (≥ 70 years) was 1.18 ± 0.22 and that in the younger group (< 70 years) was 1.17 ± 0.27 (Fig. 3B). The difference between groups was insignificant (*p* > 0.05). The mean volume change ratio in the male group was 1.19 ± 0.27 and that in the female group was 1.13 ± 0.17 (Fig. 3C). The difference between groups was insignificant (*p* > 0.05). The mean volume change ratio in the surgery + radiotherapy group was 1.21 ± 0.16 and that in surgery only group was 1.16 ± 0.28 (Fig. 3D). The difference between groups was insignificant (*p* > 0.05). The mean volume change ratio in the under 3 months group was 1.13 ± 0.27 and that in the over 3 months group was 1.21 ± 0.23 (Fig. 3E). The difference between groups was insignificant (*p* > 0.05). The mean volume change ratio in the left side group was 1.14 ± 0.26 and that in the right side group was 1.22 ± 0.22 (Fig. 3F). The difference between groups was insignificant (*p* > 0.05).

## Discussion

In this study, the size of the contralateral submandibular gland was significantly increased post-operatively after the ipsilateral submandibular gland extirpation (Fig. 3A). This increase was also noted in the radiation therapy group, indicating that the presence or absence of radiation therapy did not affect post-operative changes in submandibular gland volume (Fig. 3D). These changes were not significantly different in gender and aging (Fig. 3B, C).

After the unilateral removal of a salivary gland, the contralateral gland may undergo compensatory hypertrophy, characterized by increased proliferation and enlargement of acinar cells [11, 12]. This observation is consistent with our findings (Fig. 3A). If compensatory hypertrophy and the subsequent increase in saliva flow do not occur, xerostomia may develop. Various approaches are available for managing post-surgical xerostomia. Although artificial saliva can be used, its effects are often short-lasting [13]. Another option is the submandibular gland transfer, which involves relocating the contralateral submandibular gland to the submental area. This method has been reported to be more effective than pilocarpine [14]. However, this procedure may not be feasible for oral cancer patients, as it can lead to a loss of facial symmetry [15]. An alternative approach involves relocating the gland near the parotid area, which has shown success in avoiding radiation exposure [14]. Recently, intensity-modulated radiotherapy (IMRT) has been employed to spare the parotid gland, though there is limited literature on its use for submandibular gland sparing [4]. The effect of IMRT could be a favorable outcome factor [16]. Despite the advancements in dose-reducing IMRT, new technologies are still needed to better preserve saliva production and improve the overall quality of life for patients [9].

As individuals age, salivary glands naturally undergo atrophy, leading to a reduction in both size and function. This atrophy can potentially limit the capacity for compensatory hypertrophy in the remaining gland after one is removed. In contrast, younger patients may have a greater capacity for glandular compensation due to more robust cellular regeneration and overall physiological resilience. In this study, there was no significant difference in post-operative volume change between the groups aged over 70 and those under 70 (Fig. 3B). Aging significantly influences salivary flow, with the maximum increase in salivary flow rate observed in individuals aged 20–29 years [17]. Additionally, from birth to early adulthood, all salivary glands grow consistently in size [18]. However, as people age, studies have shown that increased cell death and reduced salivary function are major contributors to xerostomia in elderly individuals [19]. The median age in this study was 70 years, and much younger individuals were not included, which might explain the insignificant differences observed between the age groups.

In this study, the volume change ratio in males was larger than that in females; however, the difference between the groups was not statistically significant (Fig. 3C). Contrarily, a study conducted in Nepal observed that the volume of salivary glands in females is generally greater than in males, though this difference was also not significant [20]. Regarding salivary flow rates, males exhibited higher rates compared to females under both unstimulated and stimulated conditions [17]. Although this does not directly address gland size, another study found that female prisoners had a higher prevalence of salivary gland diseases compared to male prisoners [21]. Age and gender differences have a significant effect on salivary gland functions which is more apparent in women than in men [17, 22, 23]. These findings suggest that while there are observed differences in salivary gland size and function between genders, the evidence remains inconsistent and inconclusive. Further research is required to better understand these potential anatomical or physiological differences.

Radiation therapy is known to have a significant impact on salivary gland size and function [24]. The exposure of salivary glands to radiation, particularly during treatments for head and neck cancers, can induce atrophy, leading to a reduction in glandular size and a marked decrease in saliva production [25]. This radiation-induced atrophy occurs due to damage to the acinar cells, which are responsible for saliva production, as well as fibrosis and vascular changes within the gland [26]. The extent of atrophy and the consequent reduction in salivary flow can vary depending on factors such as the dose of radiation, the specific glands targeted, and the duration of the treatment [27]. Over time, this atrophy can lead to chronic xerostomia, significantly affecting a patient’s quality of life [25]. In some cases, the damage may be irreversible, with little to no recovery of glandular function. In studies examining post-radiation therapy patients, a significant reduction in salivary gland volume has been observed, confirming the deleterious effects of radiation on these glands [28]. The loss of glandular size and function after radiation therapy highlights the need for protective strategies or interventions to mitigate these effects and preserve salivary gland function. However, there was no significant difference in post-operative salivary gland volume change in the presence of post-operative radiation therapy in this study (Fig. 3D). This might be due to well-organized protective strategies or interventions to mitigate these effects and preserve salivary gland function.

Compensatory increases in saliva flow following salivary gland extirpation have been reported in a few clinical studies [29, 30]. However, this phenomenon is more commonly observed in animal studies [31, 32]. If compensatory salivation occurs, the extent of this change may vary depending on the observation period. In this study, the mean volume change ratio in the group observed for less than 3 months was slightly lower than in the group observed for more than 3 months, but this difference was not statistically significant (Fig. 3E). This suggests that compensatory gland hyperplasia occurs in the early period following unilateral salivary gland removal and is maintained thereafter. Previous reviews have shown that removal of the submandibular gland significantly reduces unstimulated saliva production [3]. Unfortunately, salivary flow was not measured in this study, so the relationship between compensatory volume increase in the submandibular gland and saliva flow remains undetermined. Additionally, a study conducted in Nepal reported slight size variations between the right and left submandibular glands: the mediolateral width of the right gland was slightly greater, while the left gland had a slightly larger volume [20]. In contrast, this study found no significant volume difference between the right and left submandibular glands (Fig. 3F).

The limitations of this study should be acknowledged. First, the sample size of oral cancer patients who underwent ipsilateral neck dissection was small, and the inclusion of younger patients was particularly limited. Although oral cancer is more prevalent in older populations, the potential for compensatory hypertrophy may vary with age, which could influence the study’s conclusions. Including a more diverse age range, particularly younger patients, might yield different outcomes. Additionally, this was a single-center study, which limits the generalizability of the findings. A multicenter study would be necessary to validate these results across different populations and settings. Second, the study was retrospective in nature, which inherently carries certain biases and limitations in data collection and analysis. Future research should focus on prospective studies to provide more robust and controlled data, allowing for a clearer understanding of the factors influencing compensatory hypertrophy and post-surgical outcomes. Third, this study did not include functional assessments such as measuring salivary flow rates or conducting radioisotope studies, which would have provided valuable insights into the functional outcomes of compensatory hypertrophy. These functional analyses are crucial for understanding the clinical significance of the morphological changes observed and should be incorporated into future research. Fourth, different types of radiotherapy may affect post-operative salivary function. In this study, most patients were referred to regional radiotherapy centers where they received IMRT. IMRT delivers precise radiation doses to the tumor or specific areas within the tumor while minimizing exposure to surrounding healthy tissues, such as the salivary glands, thereby reducing the risk of xerostomia [9, 15]. In contrast, other types of radiotherapy, such as 3D conformal radiotherapy or high-dose-rate brachytherapy, are more likely to induce xerostomia [33]. Due to the limited number of patients who received post-operative radiotherapy in this study, we were unable to evaluate the differences in post-operative salivary gland volume changes based on the type of radiotherapy received.

## Conclusion

This study demonstrates a statistically significant increase in the volume of the contralateral submandibular gland following unilateral gland excision in oral cancer patients, suggesting compensatory hypertrophy. These findings underscore the importance of considering potential morphological adaptation in the contralateral gland during post-operative care and surgical planning. While factors such as age, gender, and adjuvant radiotherapy did not significantly influence the volume change in this study, the observed hypertrophy has several clinical implications.

Understanding that the contralateral submandibular gland may undergo compensatory hypertrophy can help guide surgeons in preserving the function and blood supply of the remaining gland, particularly in cases involving bilateral surgeries or extensive neck dissection. This awareness may encourage more conservative surgical approaches or the use of alternative treatments to minimize the risk of xerostomia. Furthermore, for patients requiring post-operative radiotherapy, radiation fields can be tailored to spare the contralateral gland as much as possible to reduce the risk of radiation-induced xerostomia. By incorporating these considerations into patient consultations and treatment planning, healthcare providers can better preserve salivary function and improve the quality of life for oral cancer patients.

## Data Availability

Data sharing is not applicable to this article since no dataset was generated or analyzed during the current study.

## Abbreviations

3D: 3-Dimensional
IMRT: Intensity-modulated radiotherapy
RT: Radiotherapy
